# Efficacy, immunogenicity and safety of respiratory syncytial virus prefusion F vaccine: systematic review and meta-analysis

**DOI:** 10.1186/s12889-024-18748-8

**Published:** 2024-05-06

**Authors:** Yi Pang, Haishan Lu, Demin Cao, Xiaoying Zhu, Qinqin Long, Fengqin Tian, Xidai Long, Yulei Li

**Affiliations:** 1 Youjiang Medical University for Nationalities, Baise, China; 2 Clinicopathological Diagnosis & Research Center, The Affiliated Hospital of Youjiang Medical University for Nationalities, Baise, China; 3 Key Laboratory of Tumor Molecular Pathology of Guangxi Higher Education Institutes, Baise, China

**Keywords:** Respiratory syncytial virus, Prefusion F vaccine, Efficacy, Immunogenicity, Safety

## Abstract

**Objective:**

A notable research gap exists in the systematic review and meta-analysis concerning the efficacy, immunogenicity, and safety of the respiratory syncytial virus (RSV) prefusion F vaccine.

**Methods:**

We conducted a comprehensive search across PubMed, Embase, the Cochrane Central Register of Controlled Trials, and ClinicalTrials.gov to retrieve articles related to the efficacy, immunogenicity, and safety of RSV prefusion F vaccines, published through September 8, 2023. We adhered to the Preferred Reporting Items for Systematic Reviews and Meta-Analyses guidelines.

**Results:**

A total of 22 randomized controlled trials involving 78,990 participants were included in this systematic review and meta-analysis. The RSV prefusion F vaccine exhibited a vaccine effectiveness of 68% (95% CI: 59–75%) against RSV-associated acute respiratory illness, 70% (95% CI: 60–77%) against medically attended RSV-associated lower respiratory tract illness, and 87% (95% CI: 71–94%) against medically attended severe RSV-associated lower respiratory tract illness. Common reported local adverse reactions following RSV prefusion F vaccination include pain, redness, and swelling at the injection site, and systemic reactions such as fatigue, headache, myalgia, arthralgia, nausea, and chills.

**Conclusions:**

Our meta-analysis suggests that vaccines using the RSV prefusion F protein as antigen exhibit appears broadly acceptable efficacy, immunogenicity, and safety in the population. In particular, it provides high protective efficiency against severe RSV-associated lower respiratory tract disease.

**Supplementary Information:**

The online version contains supplementary material available at 10.1186/s12889-024-18748-8.

## Introduction

Respiratory syncytial virus (RSV), discovered in 1956, is a negative-sense single-stranded RNA virus belonging to the *Pneumonaviridae* family. RSV is highly contagious and represents a major burden of respiratory disease worldwide, causing severe and even fatal respiratory infections and bronchiolitis, especially in the elderly (≥ 65 years), young children (< 5 years), and those with underlying chronic diseases (e.g., pulmonary and circulatory diseases) [1]. In 2019, globally, there were 33 million events of RSV-associated acute lower respiratory tract infection (uncertainty range, 2.54 to 446 million) and 1.01 million total RSV-attributable deaths (84 500 to 125 200) in young children [2].

There has been a long road with multiple obstacles to developing a safe and effective RSV vaccine. Earlier vaccines provided insufficient protection as they used the post-F conformation as the vaccine antigen. This is because multiple unique antigenic sites are exposed on the surface of the F protein before RSV fuses with the host cell membrane. Following fusion, the F protein adopts a very different confirmation in which several antigenic sites are no longer exposed [3]. Thus, the stabilization of the pre-F conformation has made it possible to develop effective subunit vaccines [4]. On May 3, 2023, the U.S. Food and Drug Administration (FDA) approved the world’s first RSV vaccine (developed by GSK) and on May 31, 2023, the Pfizer vaccine, both for adults older than 60 years of age. Both vaccines use a prefusion stable variant of the F protein. RSV prefusion F vaccine has become a hot spot in the research of vaccines against RSV. A large number of clinical studies have investigated its protective efficacy. However, to date, no systematic reviews have been performed on the efficacy, immunogenicity and safety of RSV prefusion F vaccine. In this review, we compared the protective efficacy, antibody titer levels, and adverse reaction profiles of different RSV prefusion F vaccines between immunized individuals and controls.

## Methods

This systematic review adheres to the Preferred Reporting Items for Systematic Reviews and Meta-Analyses (PRISMA) guidelines [5].

### Search strategy

In September 2023, in accordance with the study protocol, we conducted searches across several databases, including Medline via PubMed, Embase, Cochrane Central Register of Controlled Trials (CENTRAL), and ClinicalTrials.gov, to identify articles published up to September 8, 2023. The following MeSH (Medical Subject Heading) terms and search terms were used: (“Respiratory Syncytial Viruses or RSV”) AND (“vaccine or vaccination or efficacy or adverse event”).

### Eligibility criteria

The inclusion criteria included: (1) individual study populations being at least twenty cases; (2) the use of prefusion F protein as an immunogen is explicitly stated; (3) clinical trials in human subjects have been published. No language restrictions were imposed on the publications. To enhance the validity of the data, we excluded non-peer-reviewed articles from preprint databases.

### Study selection

In this review, we employed a two-stage approach for screening, initially assessing titles and abstracts followed by full-text articles. Two researchers independently reviewed each title, abstract, and full text, with any discrepancies resolved through consensus with a third researcher. The efficacy of the vaccines were assessed on three endpoints. First, the efficacy of the vaccine in preventing RSV-associated acute respiratory illness which was defined as the ability of the vaccine to prevent RT-PCR-confirmed RSV infection within seven days of acute respiratory illness symptom onset. Second, the efficacy of the vaccine in preventing medically attended RSV-associated lower respiratory tract illness which was defined as at least two symptoms or signs of acute respiratory infection lasting at least 24 h (cough, abnormal breathing, fever, lethargy, or any other respiratory symptom of concern). Third, the efficacy of the vaccine in preventing medically attended severe RSV-associated lower respiratory tract illness which was defined as tachypnea (respiratory rate ≥ 70 breaths per minute in infants younger than two months [60 days] of age or ≥ 60 breaths per minute in those between two months and 12 months of age); SpO2 < 93% while the infant was breathing ambient air; use of oxygen delivered through a high-flow nasal cannula or mechanical ventilation; admission to an intensive care unit for more than 4 h; and unresponsiveness or unconsciousness. The efficacy of the RSV vaccine was based on assessing its efficacy during the first RSV season (about 6 months) after vaccination. All the efficacy endpoints were considered if they occurred at least seven days after the full regimen of the vaccine.

### Data extraction

Two researchers extracted data using a predefined extraction form. Subsequently, all key extracted data underwent review and quality checking by the same two researchers at the conclusion of the data extraction phase. Data on study characteristics encompassed information regarding the setting, primary and secondary outcomes, study design, sample size, and exclusion and inclusion criteria. Participant data included details such as sex, age, and relevant medical history, including disease and treatment history. Intervention-related data consisted of the vaccine type and brand, dosing schedule, the number of participants receiving each type and brand of vaccine, and the median or mean interval between doses. Data pertaining to immunogenicity results included details such as the assay type, the specific antibody measured, T cell response, the method of measurement, intervals of sample collection, and the number of measurements conducted.

### Risk of bias assessment

Two investigators independently evaluated the risk of bias in the included studies based on critical criteria, including random sequence generation, allocation concealment, blinding of participants, personnel, and outcomes, incomplete outcome data, selective outcome reporting, and other potential sources of bias, following the methods recommended by The Cochrane Collaboration. The risk of bias graph was generated using Revman 5.4 software. The following judgments were used: low risk, high risk, or unclear. Authors resolved disagreements by consensus and further article review if necessary.

### Data analysis

We used RevMan 5.4 statistical software to pool dichotomous outcomes, with the risk ratio (RR) and its 95% confidence interval (CI) as the effect measures. RR < 1 implies a lower risk in the vaccinated group compared to the control group, and *P* < 0.05 indicates that this difference is statistically significant. The *I*^2^ statistic was used to estimate the level of heterogeneity, and significant heterogeneity was considered when the *I*^2^ value was > 50%. Vaccine efficacy was calculated using the fixed effects RR. This study applied the accepted statistical vaccine efficacy formula, (1 − RR) ×100, for calculating the pooled vaccine efficacy from the pooled RR. We conducted visual examinations of funnel plots and utilized Egger’s test to assess potential publication bias. Additionally, we employed the trim-and-fill analysis to evaluate the effect of publication bias on the pooled effect size estimates. Influence analysis, which constitutes a form of sensitivity analysis, was performed to identify the impact of individual studies on the combined estimates.

## Results

### Study selection and study characteristics

**Fig. 1:**
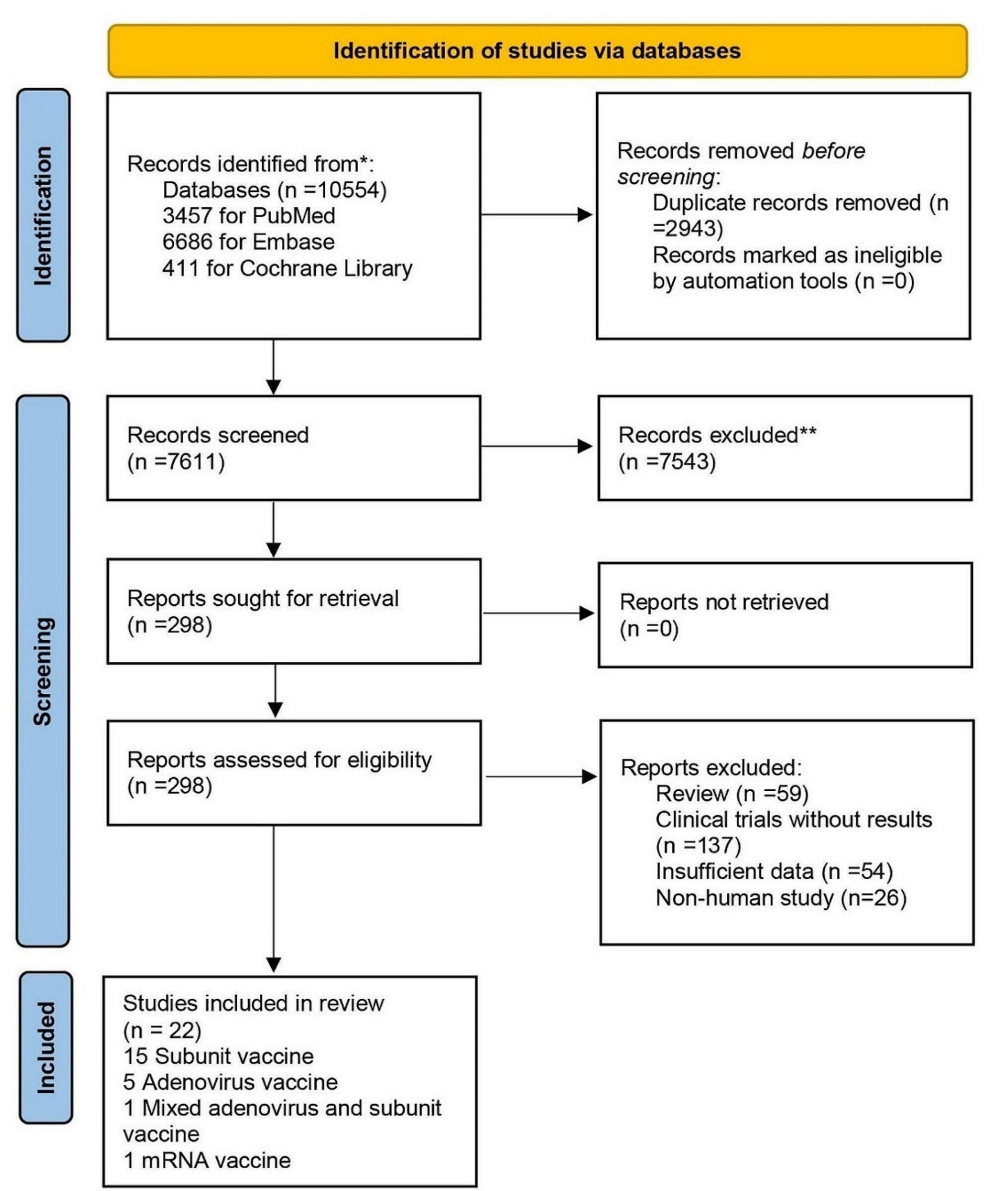
Flowchart of study selection

A total of 10,554 records were initially retrieved from the database. After screening titles and abstracts, we evaluated 298 full texts of potentially eligible reports; a total of 22 articles were included, involving 78,990 participants (Fig. 1) [6–27]. Of the 22 eligible studies, eight (36%) studies were analyzed to evaluate the efficacy of RSV prefusion F vaccines, 20 (91%) studies were analyzed to evaluate immunogenicity, and 22 (100%) studies were analyzed to evaluate safety (Table 1). The included studies reported data for four vaccine types: 15 (68%) for subunit vaccines, five (23%) for adenovirus vaccines, one (4%) for mixed adenovirus and subunit vaccines, and one (4%) for mRNA vaccines. The 22 included studies involved diverse populations, with 10 involving older adults over 60 years of age, 4 involving pregnant women, 3 involving non-pregnant women, and 7 involving healthy adults. The included studies involved more than 20 countries or regions, with 11 (50%) studies being multinational, six (27%) studies from Spain, followed by two (9%) studies from Australia, and one each from Japan, Canada, and the United Kingdom. 12 (55%) of the eligible studies were observer-blinded and 10 (45%) were double-blinded.

**Table 1:** Characteristics of studies included in meta-analysis

Study	Clinical trials registration	Vaccine types	Study design	Country/ region	Study period	Age	No. of participants	Controls	Outcomes
Walsh et al., 2023	NCT05035212	RSVpreF (Subunit vaccine)	Phase III, randomized, double-blind, multicenter, placebo-controlled study	Argentina, Canada, Finland, Japan, the Netherlands, South Africa, and the United States	August 31, 2021-July 14, 2022	≥ 60 years	34,284	17,069	Efficacy and safety
Papi et al., 2023	NCT04886596	RSVPreF3 OA (Subunit vaccine)	Phase III, randomized, observer-blind, placebo-controlled, multi-country study	Australia, Belgium, Canada, Estonia, Finland, Germany, Italy, Japan, Korea, Mexico, New Zealand, Poland, Russian Federation, South Africa, Spain, United Kingdom, and the United States	May 25, 2021-January 31, 2022	≥ 60 years	24,966	12,499	Efficacy and safety
Leroux-Roels et al., 2023	NCT03814590	RSVPreF3 (Subunit vaccine)	Phase I/II, randomized, observer-blind, placebo-controlled study	Belgium and the United States	January 21, 2019-February 23, 2021	18–40 years;60–80 years	1049	112	Safety and Immunogenicity
Kotb et al., 2023	NCT04090658	RSVPreF3/AS01B (Subunit vaccine)	Phase I, randomized, observer-blind, placebo-controlled study	Japan	September 2019-December 2020	60–80 years	40	20	Safety and Immunogenicity
Kampmann et al., 2023	NCT04424316	RSVpreF (Subunit vaccine)	Phase III, randomized, double-blind, placebo-controlled study	Argentina, Australia, Brazil, Canada, Chile, Denmark, Finland, Gambia, Japan, Korea, Mexico, Netherlands, New Zealand, Philippines, South Africa, Spain, Taiwan, the United States	June 17, 2020-November 24, 2023	18–49 years, 24–36 weeks’ gestation	7358	3676	Efficacy and safety
Falsey et al., 2023	NCT03982199	Ad26.RSV.preF-RSV preF (Mixed adenovirus and subunit vaccine)	Phase IIb, randomized, double-blind, placebo-controlled, proofof-concept study	The United States	August 5, 2019-November 13, 2019	≥ 65 years	5782	2891	Efficacy, safety and Immunogenicity
Comeaux et al., 2023	NCT03502707	Ad26.RSV.preF (Adenovirus vaccine)	Phase I/IIa, randomized, double-blind, placebo-controlled study	The United States	July 9, 2018-June 30, 2022	≥ 60 years	352	40	Safety and Immunogenicity
Bebia et al., 2023	NCT04126213	RSVPreF3 (Subunit vaccine)	Phase II, randomized, observer-blind, placebo-controlled study	Australia, Canada, Finland, France, New Zealand, Panama, South Africa, Spain, and the United States	November 5, 2019-May 14, 2021	18–40 years, pregnant women	211	66	Safety and Immunogenicity
Walsh et al., 2022	NCT03529773	RSVpreF (Subunit vaccine)	Phase I/II randomized, observer-blind, placebo-controlled, dose-finding study	The United States	April 2018-November 2019	18–49 years; 50–85 years	1208	104	Safety and Immunogenicity
Stuart et al., 2022	NCT03303625	Ad26.RSV.preF (Adenovirus vaccine)	Phase I/IIa, randomized, double-blind, placebo-controlled study	Finland the United Kingdom, and the United States	November 29, 2019-April 21, 2020	18–50 years; 1–2 years	48	16	Safety and Immunogenicity
Simões et al., 2022	NCT04032093	RSVpreF (Subunit vaccine)	Phase IIb, randomized, observer-blind, placebo-controlled, multicountry, proof-of-concept study	Chile, Argentina South Africa, and the United States	July 7, 2019-September 30, 2021	18–49 years, 24–36 weeks’ gestation	403	79	Efficacy, safety and Immunogenicity
Schwarz et al., 2022	NCT03674177	RSVPreF3 (Subunit vaccine)	Phase I/II, randomized, observer-blind, placebo-controlled, first-in-human study	Finland Germany, and the United States	October 2018-September 2019	18–45 years, non-pregnant women	501	126	Safety and Immunogenicity
Schmoele et al., 2022	NCT04785612	RSVpreF (Subunit vaccine)	Phase IIa, randomized, double-blind, single-center, exploratory study	Chile, Argentina South Africa, and the United States	November 10, 2020-April 8, 2021	18–49, 24–36 weeks’ gestation	70	35	Efficacy, safety and Immunogenicity
Sadoff et al., 2022	NCT03334695	Ad26.RSV.preF (Adenovirus vaccine)	Phase IIa, randomized, double-blind, placebo-controlled study	The United Kingdom	August 2, 2017-November 27, 2018	18–50 years	63	32	Efficacy, safety and Immunogenicity
Peterson et al., 2022	NCT04071158	RSVpreF (Subunit vaccine)	Phase Iib, randomized, observer-blind, placebo-controlled, multicenter study	The United States	October-December, 2019	18–45 years, non-pregnant women	709	141	Safety and Immunogenicity
Baber et al., 2022	NCT03572062	RSVpreF (Subunit vaccine)	Phase I/II, randomized, observer-blind, placebo-controlled, dose-finding study	Australia	April 29, 2019-August 19, 2020	65–85 years	317	/	Safety and Immunogenicity
Sadoff et al., 2021	NCT03339713	Ad26.RSV.preF (Adenovirus vaccine)	Phase IIa, randomized, double-blind, placebo-controlled, parallel-group study	The United States	December 7, 2017-July 23, 2018	≥ 60 years	180	90	Safety and Immunogenicity
Aliprantis et al., 2021	/	mRNA-1777 (mRNA vaccine)	Phase I, randomized, partially double-blind, placebo-controlled, first-in-human, dose-escalation study	Australia	November 2016-May 2019	18–49 years	242	45	Safety and Immunogenicity
Williams et al., 2020	NCT02926430	Ad26.RSV.preF (Adenovirus vaccine)	Phase I, randomized, double-blind, placebo-controlled study	The United States	November 8, 2016-May 14, 2018	≥ 60 years	73	24	Safety and Immunogenicity
Schwarz et al., 2019	NCT02956837	RSV-PreF (Subunit vaccine)	Phase II, randomized, observer-blind, multicenter study	Belgium, Estonia, France, and Germany	November 2016-February 2018	18–45 years	406	102	Safety and Immunogenicity
Beran et al., 2018	NCT02360475/NCT02753413	RSV-PreF (Subunit vaccine)	Randomized, observer-blinded, controlled study	Australia, the Czech Republic Germany, and the United States,	March 2015-June 2016	18–45 years, non-pregnant women	600	175	Safety and Immunogenicity
Langley et al., 2017	NCT01905215	RSV-PreF (Subunit vaccine)	Phase I, randomized, observer-blind, controlled, first in-humans study	Canada	July 22, 2013-March 16, 2015	18–44 year, men	128	33	Safety and Immunogenicity

### Risk of bias assessment of included studies

**Fig. 2:**
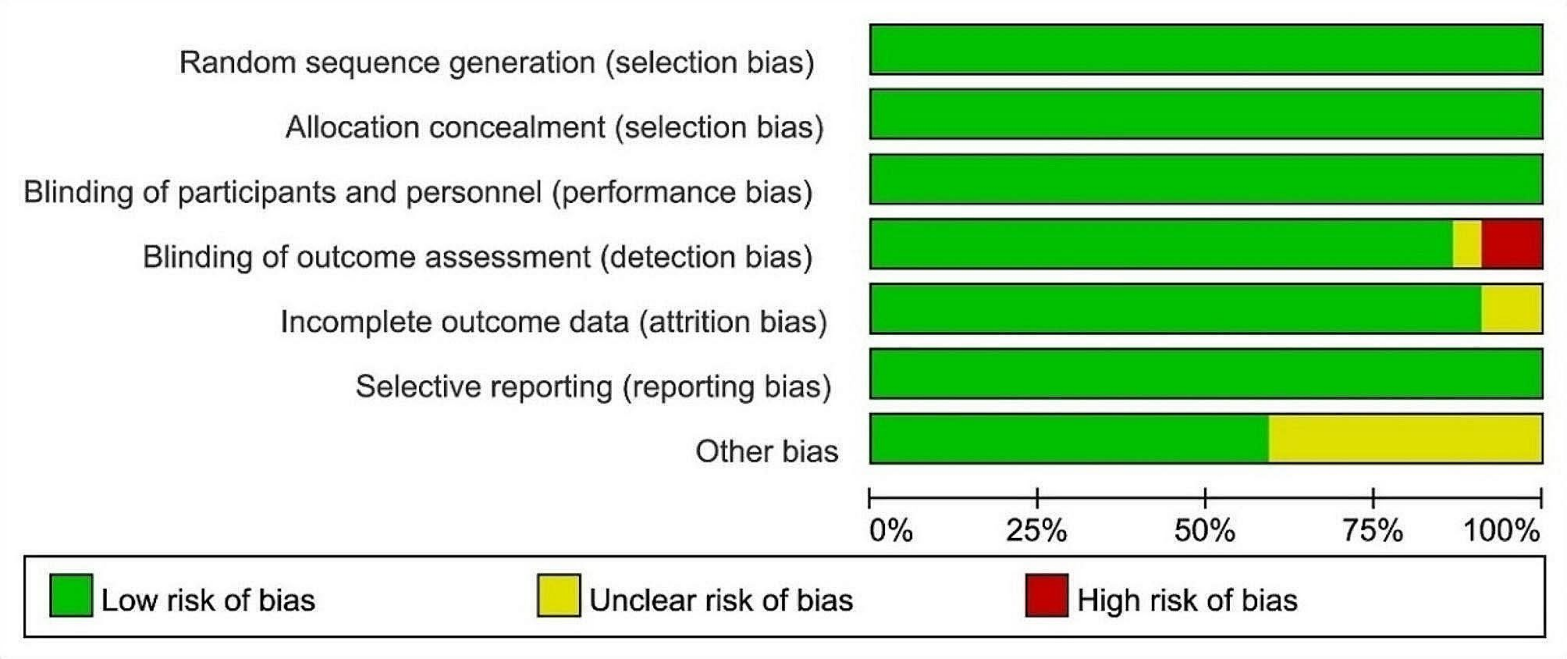
Risk of bias graph: review authors’ judgements about each risk of bias item presented as percentages across all included studies

Twenty-two studies used Cochrane collaboration tools for independent risk of bias assessment, only two studies had high risk in blinding of outcome assessment, and most studies were low risk in all evaluated domains (Fig. 2). Overall, all of these included studies had a low risk of bias, with blinding and other biases in outcome assessment being the main risk factors.

### Efficacy of RSV prefusion vaccine

**Fig. 3:**
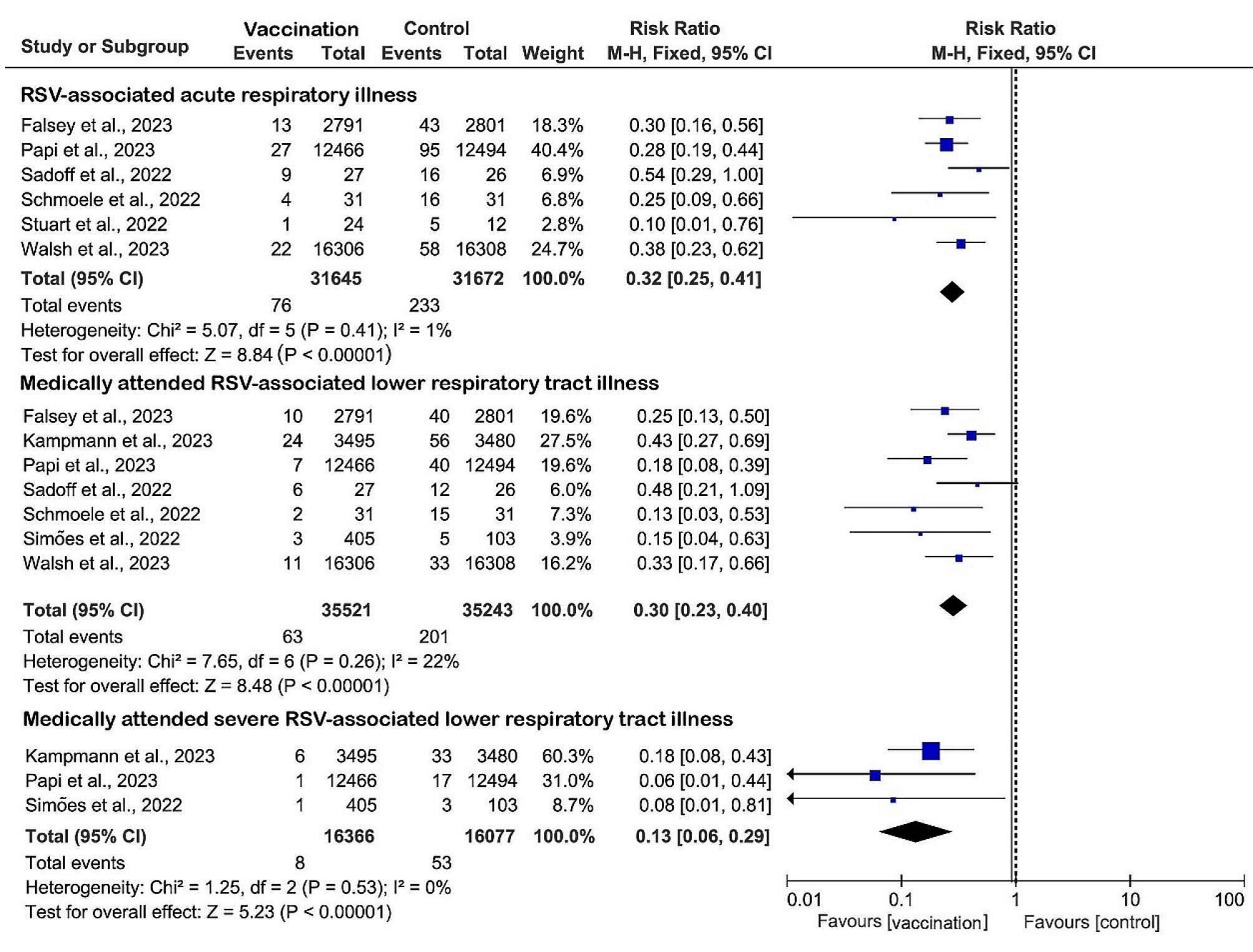
Vaccine efficacy compared with placebo calculated using the Mantel–Haenszel fixed effects model

Six (27%) studies were included to evaluate the efficacy of RSV prefusion vaccine in the prevention of RSV-associated acute respiratory illness. Data from 31,645 vaccinated patients compared with 31,672 controls showed a significant pooled risk reduction in the vaccinated group, with a RR of 0.32 (95% CI: 0.25 to 0.41, *I*^2^ = 1%) and an overall vaccine efficacy of 68% (95% CI: 59–75%) (Fig. 3). A total of seven (32%) studies assessing the efficacy of vaccination against medically attended RSV-associated lower respiratory tract illness with data from 35,521 vaccinated versus 35,243 controls showed similarly significant pooled risk reductions in vaccinated groups, with a RR of 0.30 (RR 0.30, 95% CI: 0.23 to 0.40, *I*^2^ = 22%). Three (14%) studies reported the lowest RR (RR 0.13, 95% CI: 0.06 to 0.29, *I*^2^ = 0%) and minimal heterogeneity in severe RSV-associated lower respiratory tract illness requiring medical attention in the group that received the RSV prefusion F vaccines, with an overall vaccine efficacy of 87% (95% CI: 71–94%). When sensitivity analyses were performed, the heterogeneity of the pooled effects of the results did not change substantially after retaining only subunit vaccines, indicating that our results are robust and reliable.

### Immunogenicity of RSV prefusion vaccine

Following the inclusion criteria, 20 studies (91%) on the immunogenicity of RSV prefusion F vaccines were included in this systematic review article (Table 2). There was a significant increase in neutralizing antibody titers against RSV-A in all studies, with a maximum increase of more than 20-fold from baseline. The neutralizing antibody titer against RSV-B was also significantly increased at about one month after immunization, with an increase of more than 1.4-fold compared with baseline. Seven studies examined T cell responses after vaccine immunization simultaneously, and the results showed that mixed adenovirus and subunit vaccine produced the strongest cellular immune responses, with up to 13-fold increase in interferon-γ secretion compared with baseline.

**Table 2:** Humoral and cellular immune responses following vaccination

Study	Vaccine types	Immunoassay days	RSV-A nAb GMFI	RSV-B nAb GMFI	RSV pre-F binding antibodies	RSV post-F binding antibodies	T cell response*
Papi et al., 2023	RSVPreF3 OA (Subunit vaccine)	D31	10.2	8.5	13	/	/
Leroux-Roels et al., 2023	RSVPreF3 (Subunit vaccine)	D31	5.6–13.7	9.2–10	7.2–13.5	/	/
Kotb et al., 2023	RSVPreF3/AS01B (Subunit vaccine)	D30	7.3	8.4	12.8	/	/
Falsey et al., 2023	Ad26.RSV.preF-RSV preF (Mixed adenovirus and subunit vaccine)	D15	12.1	9.4	8.6	/	13
Comeaux et al., 2023	Ad26.RSV.preF (Adenovirus vaccine)	D29	2.7–10.5	/	2.1–13.8	/	2.8–9.7
Bebia et al., 2023	RSVPreF3 (Subunit vaccine)	D31	12.7–14.9	10.6–13.2	13.4–17.7	/	/
Walsh et al., 2022	RSVpreF (Subunit vaccine)	D31	10.6–16.9	10.3–19.8	16.4–30.6	/	/
Stuart et al., 2022	Ad26.RSV.preF (Adenovirus vaccine)	D29	13.3	27.9	19.9	8.9	/
Simões et al., 2022	RSVpreF (Subunit vaccine)	/	11.0-15.1	13.7–17.5	/	/	/
Schwarz et al., 2022	RSVPreF3 (Subunit vaccine)	D31	6.26–7.95	/	6.8–14.0	/	/
Schmoele et al., 2022	RSVpreF (Subunit vaccine)	D28	20.5	20.3	/	/	/
Sadoff et al., 2022	Ad26.RSV.preF (Adenovirus vaccine)	D28	5.8	/	6.8	4.2	/
Peterson et al., 2022	RSVpreF (Subunit vaccine)	D31	14.1	14.6	/	/	/
Baber et al., 2022	RSVpreF (Subunit vaccine)	D31	4.8–11.6	4.5–14.1	6.4–14.3	/	1.1–1.8
Sadoff et al., 2021	Ad26.RSV.preF (Adenovirus vaccine)	D28	2.8–3.1	/	2.3–2.6	2.0-2.1	/
Aliprantis et al., 2021	mRNA-1777 (mRNA vaccine)	D29	2.5–4.3	/	1.7–4.5	/	2.2–3.7
Williams et al., 2020	Ad26.RSV.preF (Adenovirus vaccine)	D29	1.6–2.1	1.7-2.0	1.5–1.7	/	2.1–2.4
Schwarz et al., 2019	RSV-PreF (Subunit vaccine)	D30	3.75–4.36	2.36–2.76	5.86–6.74	/	/
Beran et al., 2018	RSV-PreF (Subunit vaccine)	D30	3.1–3.9	/	25.7–38.2	/	/
Langley et al., 2017	RSV-PreF (Subunit vaccine)	D30	1.28–2.92	1.40–2.23	2.5–4.2	/	/

*T cell responses were measured with an interferon-γ enzyme-linked immunosorbent spot assay. nAb, neutralizing antibody; GMFI, geometric mean fold increase

### Safety of RSV prefusion vaccine

**Fig. 4:**
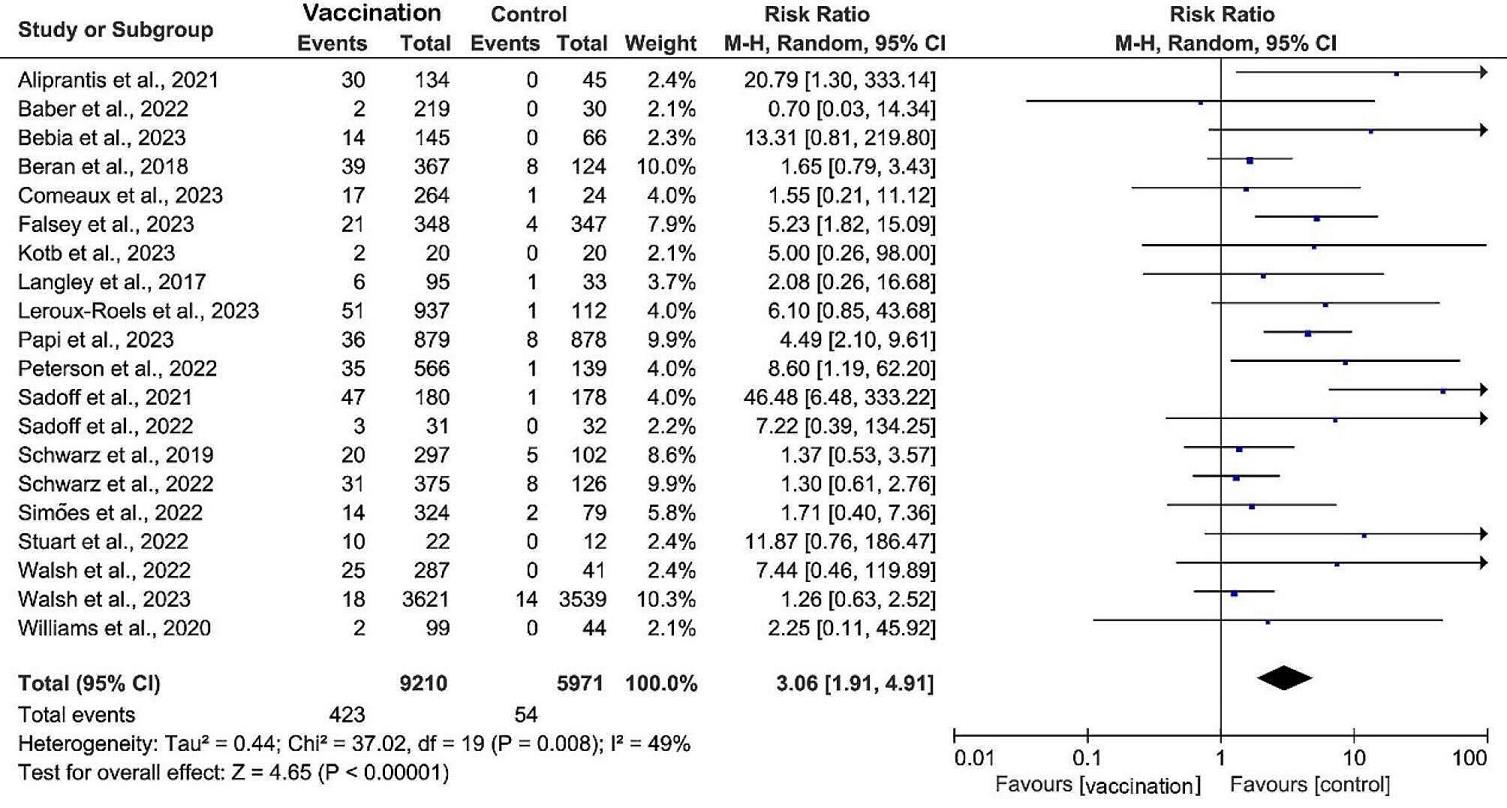
Incidence of grade ≥ 3 adverse events among the vaccination versus the control group

The safety profiles of 22 studies were reviewed, and adverse effects of RSV prefusion F vaccination included local reactions such as pain, redness, and swelling at the vaccination site and systemic reactions such as fatigue, headache, Myalgia, joint pain, nausea, and chills (Table 3). The subunit vaccine had the lowest risk of local and systemic adverse reactions, with RR of 2.79 (95% CI: 1.47 to 6.00, *I*^2^ = 77%) and 1.24 (95% CI: 0.95 to 1.63, *I*^2^ = 74%), respectively, and the risk of serious adverse events (grade ≥ 3) was also the lowest (RR 2.11, 95% CI: 1.41 to 3.15, *I*^2^ = 25%) (Fig. 4; Table 3). Redness was the predominant local reaction observed among recipients of the subunit vaccine (RR 4.77, 95% CI: 3.08 to 7.38, *I*^2^ = 41%). Conversely, pain at the injection site was the most common local symptom in the mRNA vaccine (RR 40.63, 95% CI: 5.85 to 282.44). Myalgia (RR 3.96, 95% CI: 2.35 to 6.66, *I*^2^ = 29%), nausea (RR 3.74, 95% CI: 0.83 to 16.9, *I*^2^ = 75%) and chill (RR 7.37, 95% CI: 4.20 to 12.94, *I*^2^ = 0%) were the most common symptoms reported in recipients of adenovirus vaccine. Of note, the mRNA vaccine exhibited the highest risk of adverse effects graded as 3 or higher (RR 20.79, 95% CI: 1.30 to 333.14). No RSV prefusion F vaccine-related deaths were recorded in these studies.

**Table 3:** Incidence of adverse events among the vaccination versus the control group

Adverse events	Vaccine type	No. of studies	Reaction/total		RR (95%CI)	Heterogeneity I^2^ (%)	Test of effect size (*p* value)
			Vaccination	Control			
Local adverse events (any)	Overall	11	1239/5067	365/4363	3.43 [2.38, 4.96]	83	< 0.00001
	Subunit vaccine	4	614/4000	262/3671	2.97 [1.47, 6.00]	77	0.002
	Adenovirus vaccine	5	362/585	67/300	3.15 [1.95, 5.10]	62	< 0.00001
	Mixed adenovirus and subunit vaccine	1	132/348	29/347	4.54 [3.12, 6.59]	/	< 0.00001
	mRNA vaccine	1	131/134	7/45	6.28 [3.18, 12.42]	/	< 0.00001
Systemic adverse events (any)	Overall	11	1814/5067	1136/4353	1.68 [1.25, 2.26]	90	0.0005
	Subunit vaccine	4	1242/4000	981/3671	1.24 [0.95, 1.63]	74	0.12
	Adenovirus vaccine	5	328/585	82/290	1.65 [1.08, 2.50]	75	0.02
	Mixed adenovirus and subunit vaccine	1	144/348	57/347	2.52 [1.93, 3.29]	/	< 0.00001
	mRNA vaccine	1	100/134	16/45	2.10 [1.40, 3.15]	/	0.0003
Injection site pain	Overall	22	4917/12,817	957/9621	3.72 [2.42, 5.74]	97	< 0.00001
	Subunit vaccine	15	4317/11,804	871/8939	3.32 [1.94, 5.69]	98	< 0.0001
	Adenovirus vaccine	5	359/585	61/290	3.44 [2.41, 4.91]	25	< 0.00001
	Mixed adenovirus and subunit vaccine	1	120/348	24/347	4.99 [3.30, 7.53]	/	< 0.00001
	mRNA vaccine	1	121/134	1/45	40.63 [5.85, 282.44]	/	0.0002
Redness	Overall	22	748/12,871	97/9621	4.48 [3.23, 6.20]	24	< 0.00001
	Subunit vaccine	13	677/11,804	86/8939	4.77 [3.08, 7.38]	41	< 0.00001
	Adenovirus vaccine	5	17/585	1/290	3.65 [0.97, 13.72]	0	0.05
	Mixed adenovirus and subunit vaccine	1	22/348	7/347	3.13 [1.36, 7.24]	/	0.008
	mRNA vaccine	1	32/134	3/45	3.58 [1.15, 11.14]	/	0.03
Swelling	Overall	21	672/12,836	108/9588	3.01 [1.95, 4.65]	62	< 0.00001
	Subunit vaccine	12	555/11,769	80/8906	4.17 [2.52, 6.92]	52	< 0.00001
	Adenovirus vaccine	5	96/585	18/290	2.28 [0.87, 6.00]	64	0.09
	Mixed adenovirus and subunit vaccine	1	12/348	6/347	1.99 [0.76, 5.25]	/	0.16
	mRNA vaccine	1	9/134	4/45	0.76 [0.24, 2.34]	/	0.63
Fatigue	Overall	22	4395/12,871	2640/9625	1.45 [1.25, 1.69]	84	< 0.00001
	Subunit vaccine	13	3993/11,804	2536/8943	1.25 [1.09, 1.43]	79	0.001
	Adenovirus vaccine	5	240/585	52/290	2.11 [1.28, 3.48]	66	0.004
	Mixed adenovirus and subunit vaccine	1	96/348	42/347	2.28 [1.64, 3.17]	/	< 0.00001
	mRNA vaccine	1	66/134	10/45	2.22 [1.25, 3.93]	/	0.006
Headache	Overall	22	3419/12,871	1873/9625	1.55 [1.32, 1.81]	79	< 0.00001
	Subunit vaccine	13	3085/11,804	1787/8943	1.36 [1.18, 1.57]	72	< 0.0001
	Adenovirus vaccine	5	200/585	48/290	1.93 [1.22, 3.05]	59	0.005
	Mixed adenovirus and subunit vaccine	1	83/348	29/347	2.85 [1.92, 4.24]	/	< 0.00001
	mRNA vaccine	1	51/134	8/45	1.90 [1.02, 3.55]	/	0.04
Myalgia	Overall	18	2649/11,737	1123/9240	2.32 [1.80, 2.98]	85	< 0.00001
	Subunit vaccine	11	2279/10,670	1057/8558	1.85 [1.42, 2.42]	85	< 0.00001
	Adenovirus vaccine	5	216/585	31/290	3.96 [2.35, 6.66]	29	< 0.00001
	Mixed adenovirus and subunit vaccine	1	95/348	30/347	3.16 [2.15, 4.63]	/	< 0.00001
	mRNA vaccine	1	59/134	5/45	3.96 [1.70, 9.25]	/	0.001
Arthralgia	Overall	16	1373/11,244	759/8827	1.93 [1.40, 2.66]	81	< 0.0001
	Subunit vaccine	10	1209/10,525	739/8492	1.51 [1.11, 2.06]	80	0.009
	Adenovirus vaccine	5	137/585	18/290	3.43 [1.44, 8.16]	60	0.005
	mRNA vaccine	1	27/134	2/45	4.53 [1.12, 18.31]	/	0.03
Nausea	Overall	15	1260/9901	915/8230	1.39 [1.02, 1.88]	73	0.04
	Subunit vaccine	8	1127/8834	895/7548	0.99 [0.82, 1.21]	46	0.95
	Adenovirus vaccine	5	83/585	9/290	3.74 [0.83, 16.90]	75	0.09
	Mixed adenovirus and subunit vaccine	1	31/348	7/347	4.42 [1.97, 9.89]	/	0.0003
	mRNA vaccine	1	19/134	4/45	1.60 [0.57, 4.44]	/	0.37
Chill	Overall	8	271/1676	21/467	4.21 [2.06, 8.62]	55	< 0.0001
	Subunit vaccine	2	94/957	4/132	3.10 [1.22, 7.84]	0	0.02
	Adenovirus vaccine	5	159/585	12/290	7.37 [4.20, 12.94]	0	< 0.00001
	mRNA vaccine	1	18/134	5/45	1.21 [0.48, 3.07]	/	0.69
≥Grade 3	Overall	20	423/9210	54/5971	3.06 [1.91, 4.91]	49	< 0.00001
	Subunit vaccine	13	293/8132	48/5289	2.11 [1.41, 3.15]	25	0.0003
	Adenovirus vaccine	5	79/585	2/290	7.24 [1.60, 32.65]	47	0.01
	Mixed adenovirus and subunit vaccine	1	21/348	4/347	5.23 [1.82, 15.09]	/	0.002
	mRNA vaccine	1	30/134	0/45	20.79 [1.30, 333.14]	/	0.03

## Discussion

In this systematic review and meta-analysis of 22 studies, we explore for the first time the efficacy, immunogenicity, and safety of RSV prefusion F vaccine. We found that administration of the RSV prefusion F vaccine significantly reduced the risk of RSV-associated acute respiratory illness, particularly the risk of severe cases of RSV-associated lower respiratory tract illness requiring medical attention. Previous studies have found that vaccines using the fused RSV F protein as antigen, although immunogenic, do not prevent RSV-associated acute respiratory illness in the elderly, and there is no clinically identifiable patient population that may benefit from this vaccine [28]. The failure of these clinical studies has led to intensive investigation of the immune mechanism of RSV. Valuable experience has been accumulated for the development of safe and effective vaccines targeting the F prefusion protein of RSV. In eight studies involving the evaluation of vaccine efficacy, subunit vaccines appeared to provide better protection than adenovirus vaccines, but due to the limited number of studies of the two vaccines included in this study, further research remains imperative.

This study provides a comprehensive assessment of the available literature on RSV prefusion F vaccines. We found that existing subunit vaccines, adenovirus vaccines, mixed subunit and adenovirus vaccines, and mRNA vaccines were able to generate significant immune responses against RSV in vaccine recipients. The titers of neutralizing antibodies against RSV-A and RSV-B and RSV-specific ligation antibodies were significantly different among different vaccine types due to the differences in immunogenicity composition, whether they contained adjuvants or not, immunization dose, immunization times, and detection time. In our study, five studies used the ELISPOT assay to measure T-cell immune responses and showed that subunit vaccines elicited weaker T-cell responses than adenovirus vaccines, mixed subunit and adenovirus vaccines, and mRNA vaccines, which is consistent with the results of a large number of studies of COVID-19 vaccines [29, 30].

Local adverse reactions after vaccination are more common than systemic adverse reactions. For different vaccine types, subunit vaccines are significantly safer and have lower incidence of local and systemic adverse reactions. Consistent with our results, the mRNA vaccine was associated with the highest incidence of adverse reactions except for a few [31]. In addition, mRNA vaccines have a higher association with serious adverse effects than other vaccine types [32]. Myalgia, nausea, and chills were the most common symptoms reported by adenovirus vaccine recipients, findings that were also similar to those previously reported for influenza and COVID-19 vaccines [30]. In theory, these differences could be attributed to differences in the strength of the immune response to the different vaccines [33, 34], as confirmed by the efficacy and immunization results of this review.

In addition, there is concern about whether RSV vaccination can cause a potentially risky rare neurologic disorder (Guillain-Barre syndrome). While GBS is considered uncommon, it remains a significant subject of discussion in the context of vaccination. Previous research on influenza vaccination has reported an eightfold rise in the risk of GBS [35]. Similarly, investigations into COVID-19 vaccines have indicated diverse clinical associations between COVID-19 vaccination and GBS [36, 37]. It is noteworthy that, reassuringly, there is currently no observed elevated risk of GBS associated with RSV vaccination.

This study has several limitations. First, current studies of RSV vaccine protection have been based on assessments of effectiveness during the first RSV season after vaccination (approximately 6 months). There were insufficient data to evaluate the duration of efficacy and immune effects after vaccination, and whether the vaccines result in long-term adverse events, thus necessitating long-term surveillance and study for the population. Second, the study included four vaccine types, but there was considerable variation in the number of studies across vaccine types. To eliminate this effect, we performed a subgroup analysis.

In conclusion, our meta-analysis suggests that vaccines using the RSV prefusion F protein as antigen exhibit favorable efficacy, immunogenicity, and safety in the population. In particular, it provides high protective efficiency against severe RSV-associated lower respiratory tract disease.

### Electronic supplementary material

Below is the link to the electronic supplementary material.

Supplementary Material 1

## Data Availability

The original contributions presented in the study are included in the article/supplementary material. Further inquiries can be directed to the corresponding author.
