# Supplementary material for: Efficacy, immunogenicity and safety of respiratory syncytial virus prefusion F vaccine: systematic review and meta-analysis
Source: BMC Public Health. 2024 May 6;24:1244. doi: 10.1186/s12889-024-18748-8 (PMC11075318; doi:10.1186/s12889-024-18748-8)
Supplement: Supplementary file 1 — Supplementary Material 1 [file 12889_2024_18748_MOESM1_ESM.docx]

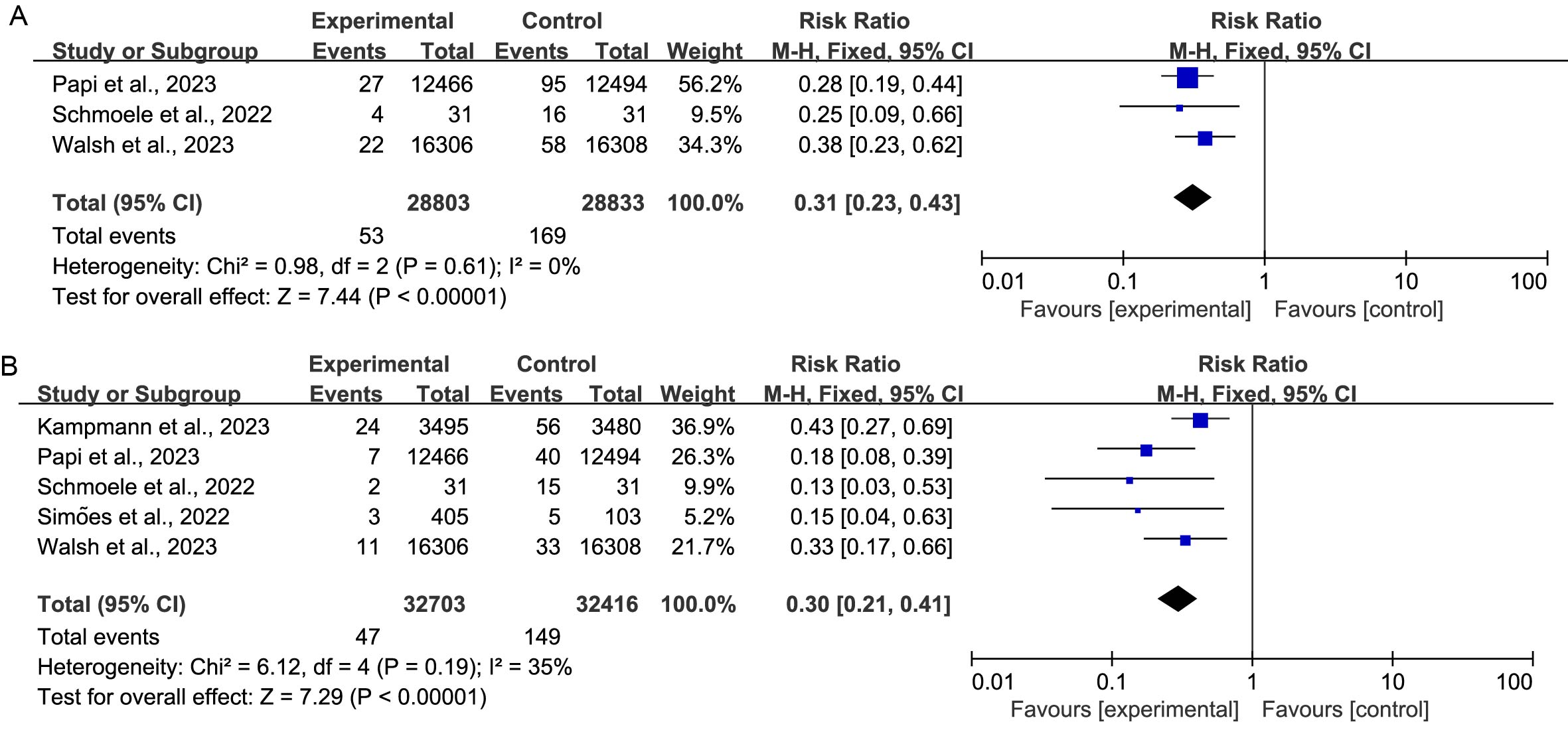


Supplementary figure 1 Sensitivity analysis of the efficacy of subunit vaccines. (A) RSV-associated acute respiratory illness. (B) Medically attended RSV-associated lower respiratory tract illness.
